# ErbB1 and ErbB3 co-over expression as a prognostic factor in gastric cancer

**DOI:** 10.1186/s40659-018-0208-1

**Published:** 2019-01-08

**Authors:** Meysam Moghbeli, Yasha Makhdoumi, Mehrdad Soltani Delgosha, Azadeh Aarabi, Ezzat Dadkhah, Bahram Memar, Abbas Abdollahi, Mohammad Reza Abbaszadegan

**Affiliations:** 1 Department of Modern Sciences and Technologies, Faculty of Medicine, Mashhad University of Medical Sciences, Mashhad, Iran; 2 Cancer Research Center, Mashhad University of Medical Sciences, Mashhad, Iran; 3 Medical Genetics Research Center, Mashhad University of Medical Sciences, Mashhad, Iran; 4 Immunology Research Center, Mashhad University of Medical Sciences, Mashhad, Iran; 5 Surgical oncology research center, Mashhad University of Medical Sciences, Mashhad, Iran; 6 Surgical oncology research center, Mashhad University of Medical Sciences, Mashhad, Iran

**Keywords:** Gastric cancer, EGFR, HER3, Prognosis, Targeted therapy

## Abstract

**Background:**

Epidermal growth factor receptor family members such as ErbB1 and ErbB3 are involved in tumor progression and metastasis. Although, there are various reports about the prognostic value of EGFR members separately in gastric cancer, there is not any report about the probable correlation between ErbB1 and ErbB3 co-expression and gastric cancer prognosis. In present study, we assessed the correlation between ErbB1 and ErbB3 co-overexpression (in the level of mRNA and protein expression) and gastric cancer prognosis for the first time.

**Methods:**

ErbB1 and ErbB3 expressions were analyzed by immunohistochemistry and real-time PCR in 50 patients with gastric cancer. Parametric correlations were done between the ErbB1 and ErbB3 expression and clinicopathological features. Multivariate and logistic regression analyses were also done to assess the roles of ErbB1 and ErbB3 in tumor prognosis and survival.

**Results:**

There were significant correlations between ErbB1/ErbB3 co-overexpression and tumor size (p = 0.026), macroscopic features (p < 0.05), tumor differentiation (p < 0.05), stage of tumor (p < 0.05), and recurrence (p < 0.05). Moreover, ErbB1/ErbB3 co-overexpression may predict the survival status of patients (p < 0.05).

**Conclusion:**

ErbB1 and ErbB3 co-overexpression is accompanied with the poor prognosis and can be used efficiently in targeted therapy of gastric cancer patients.

## Background

Gastric cancer is known as the fourth common cancer and the second leading cause of cancer-related deaths worldwide [1]. Most of the gastric cancer patients are diagnosed in advanced stages of tumor leading to a poor prognosis [2]. Tumor recurrence after the surgery is more common in tumors with advanced stages; therefore, adjuvant systemic chemotherapies have been developed to improve this problem [2]. It has been shown that the patients with advanced gastric cancer who can tolerate the side effects of chemotherapeutic treatments have real benefit in survival in comparison with the supportive care. However, the median survival time is not more than 13 months even in patients who were undergone the chemotherapeutic modalities [3]. The epidermal growth factor receptor (EGFR) family are the members of tyrosine kinase receptors that stimulate a number of signaling pathways and regulate diverse cellular processes such as proliferation, differentiation, survival, and migration [4, 5]. These signaling pathways are important in normal cellular homeostasis, therefore aberrant activation of the EGFR members can cause tumorigenesis. This protein family is comprised of four members including; ErbB1 (EGFR), ErbB2 (HER^−^2/neu), ErbB3, and ErbB4 [6]. All of these four members share a common structure; these tyrosine kinases contain an extracellular ligand-binding domain with approximately 630 amino acids and a cytoplasmic tyrosine kinase domain [7–11]. Activation of these tyrosine kinases causes autophosphorylation on specific tyrosine residues and triggers a downstream signaling cascade via the phosphoinositide-3 kinase (PI3K) and activated-Akt pathway. Therefore, ErbB family can be involved in malignant progression [12]. Except the ErbB2 that does not have a known ligand, the other members of ErbB family can be activated by specific ligands. Ligand-receptor interaction causes specific structural changes and receptor dimerization. It can be either homodimerization (ErbB2–ErbB2) or heterodimerization (ErbB1–ErbB4, ErbB2–HER3) [13]. Overexpression of ErbB receptors and their cognate ligands has been considered as one of the main causes of tumor progression [14, 15]. The ErbB1 and ErbB2 overexpression in gastric cancer is shown to be as the prognostic and efficient factors for the targeted therapy [2]. Gene amplification or EGFR overexpression have been observed in different solid tumors such as lung, colorectal, urinary bladder, breast, head and neck, esophageal, and gastric carcinomas [16–25]. ErbB3 overexpression was observed in a wide range of cancers including breast, ovary, lung, and prostate, as well as melanoma [26–30]. Although there is not any report of ErbB3 or ErbB4 overexpression in gastric cancer, ErbB3 expression is frequently seen in advanced stages of gastric cancers and is correlated with poor prognosis [31–33]. In human breast cancer, ErbB3 interacts with ErbB2 and thereby generates a potent oncogenic dimer that causes tumor progression [34]. Apart from various reports about the prognostic value of EGFR members separately in gastric cancer, there is not any report about the probable correlation between ErbB1 and ErbB3 co-expression and gastric cancer prognosis. Indeed, a panel of double EGFR markers may be more efficient in comparison with all of the regular single based markers in gastric cancer cases. In this study, ErbB1 and ErbB3 expressions in the levels of mRNA and protein were assessed for the first time in gastric cancer cases to evaluate the probable role of ErbB1 and ErbB3 co-expression in gastric cancer prognosis.

## Methods

### Tissue samples

Fresh tumor and adjacent normal tissues were obtained from 50 patients with gastric adenocarcinoma who were underwent a gastrectomy at Omid Hospital of Mashhad University of Medical Sciences. The patients did not receive any chemo-radio therapeutic treatment prior the surgery. Tissue samples were transferred to the RNA later solution immediately and stored in − 20 °C until RNA extraction. Inclusion criteria involved: no chemo–radio therapeutic treatment before the surgery and the tumor tissues were histologically examined by a pathologist to ensure that they contain at least 70% of tumor cells. Informed consent forms were obtained from the patients and protocol of study was approved by the research ethic committee.

### Immunohistochemistry

The paraffin embedded tissues were stained with hematoxylin–eosin method to be checked for the presence of tumor cells. After deparaffinization and rehydration, all of the 2 mm-thick sections were heated-pretreated with EDTA-TRIS solution (PH = 7) in a microwave oven. After washing by TBS (Biogene, Australia), endogenous peroxide activity was inhibited by H2O2 (3%, Novocastra, USA). Protein block incubation was also performed to reduce the non-specific staining. The protocol followed by incubation of slides with ErbB1 and ErbB3 monoclonal antibodies (DAKO, Denmark). After 30 min of incubation with antibody, the slides were incubated by post primary block and then by Novolink polymer for 30 min (Novolink detection system REF RE7280-K). Finally, the slides were stained with DAB chromogen and hematoxylin.

### Immunohistochemical scoring system

IHC staining was graded based on American Society of Clinical Oncology/College of American Pathology guideline [35, 36]. This criterion included, 0 = No membranous staining in tumor cells, 1+ = Weak (staining in less than 10% of cells), 2+ = intense complete staining (up to 30% of tumor cells), and 3+ = Uniform intense staining (more than 30% of tumor cells). Scores of 2+/3+ and 0/+1 were classified as over and normal expression, respectively.

### RNA extraction, cDNA Synthesis, and quantitative RT-PCR

RNA extraction from the normal and tumor tissues was performed using RNeasy Mini kit (Qiagen, Germany). RevertAid first strand cDNA synthesis kit (Fermentas, Lithuania) was also used for the mRNA reverse transcription. Quantitative Real-time PCR was performed by specific primer sets (Table 1) in Stratagene Mx-3000P real-time thermocycler (Stratagene, La Jolla, CA) using the SYBR green PCR Master Mix (Fermentas, Lithuania). The following thermal profile was applied: 10 min at 95 °C and (15 s at 95 °C, 30 s at 57 °C, and 45 s at 72 °C) 40 cycles. Data were normalized by glyceraldehyde 3-phosphate dehydrogenase (GAPDH) [37]. All experiments were performed in duplicates. A more than two-fold fluorescence intensity of mRNA expression in tumor tissue in comparison with the corresponding normal tissues was considered as over expression. Less than two-fold expression also was indicated as under expression, and the range between them was interpreted as normal expression.

**Table 1:** primer sequences for real time PCR

Primer sequence (5′ to 3′)	Amplified target	Size of amplicon (bp)
GGAGAACTGCCAGAAACTGACC	ErbB1–Exon junction 5–6	106
GCCTGCAGCACACTGGTTG	ErbB1–Exon 6	
CCCTGCCATGAGAACTGCAC	ErbB3–exon15	112
TCACTGTCAAAGCCATTGTCAGAT	ErbB3–exon17	

### Prognostic assessment

The patients were followed up 6 and 12 months after surgery by radio-oncologists. During each visit, all of patients were evaluated for recurrent disease and Karnofsky score (CA19-9) was measured through the physical examinations and diagnostic imaging. Thereafter, the prognosis was determined by oncologist based on clinical manifestation of patients.

### Statistical analysis

The statistical analysis was done using SPSS 19.0 software (SPSS, Chicago, IL). X^2^ test and Fisher exact test were used for possible association between the levels of each proteins expression and clinicopathological features. Kaplan–Meier curves were generated for overall survival and statistical significance was determined using the log-rank test. Correlation between ErbB1 and ErbB3 levels of gene expression was evaluated by Pearson’s correlation. The multivariate proportional Cox models were applied to assess the prognostic significance of HER expressions, Lauren classification, lymph node involvement, tumor invasion, and differentiation. Logistic regression model was used to assess if the ErbB1 and ErbB3 can be used as independent prediction factors of prognosis. All the statistical tests were defined significantly as a p < 0.05.

## Results

### Clinicopathological features of patients

Fifty gastric cancer patients comprising 38 (76%) male and 12 (24%) females were enrolled in the present study. Age of patients was ranged between 40 and 80 years old with mean age of 69 ± 9.3 years old. Tumor sizes were also between 3 and 10 cm with mean size of 8.2 ± 1.7 cm. Majority of tumors were located in non-cardia (38/50, 76%), positive lymph node metastasis (48/50, 96%), differentiated (30/50, 60%), in tumor stages of I/II (28/50, 56%), and with T3/4 depth of invasion (47/50, 94%). Moreover, twenty-one out of 50 (42%) patients had tumor relapse. All the clinicopathological features of patients are mentioned in Table 2.

**Table 2:** Clinicopathological characteristics of patients and their correlation with ErbB1/3 protein expression

	Total	ErbB1		*p*	ErbB3		*p*	ErbB1 and ErbB3		*p*
		Over expression	Normal		Over expression	Normal		Over expression	Normal	
Age	96 (40–80)									
Sex				0.313			0.485			0.791
Male	38 (76%)	19 (50%)	19 (50%)		16 (42.1%)	22 (57.9%)		11 (28.9%)	27 (71.1%)	
Female	12 (24%)	4 (33.3%)	8 (66.7%)		4 (33.3%)	8 (66.7%)		3 (25%)	9 (75%)	
Size	82 (30–100)									
Location				0.730			0.485			0.797
Cardia	12 (24%)	5 (41.7%)	7 (58.3%)		4 (33.3%)	8 (66.7%)		3 (25%)	9 (75%)	
Non-cardia	38 (76%)	18 (47.4%)	20 (52.6%)		16 (42.1%)	22 (57.9%)		11 (28.9%)	27 (71.1%)	
Lauren classification				0.999			0.999			0.550
Intestinal	47 (94%)	22 (46.8%)	25 (53.2%)		19 (40.4%)	28 (59.6%)		14 (29.8%)	33 (70.2%)	
Diffuse	3 (6%)	1 (33.3%)	2 (66.7%)		1 (33.3%)	2 (66.7%)		–	3 (100%)	
Macroscopy				*0.028*			*< 0.05*			*< 0.05*
Infiltrative	18 (36%)	12 (66.7%)	6 (33.3%)		17 (94.4%)	1 (5.6%)		12 (66.7%)	6 (33.3%)	
Ulceroinfiltrative	32 (64%)	11 (34.4%)	21 (65.6%)		3 (9.4%)	29 (90.6%)		2 (6.2%)	30 (93.8%)	
Differentiation				*0.028*			*< 0.05*			*< 0.05*
Poorly	20 (40%)	13 (65%)	7 (35%)		19 (95%)	1 (5%)		13 (65%)	7 (35%)	
Not-poorly	30 (60%)	10 (33.3%)	20 (66.7%)		1 (3.3%)	29 (96.7%)		1 (3.3%)	29 (96.7%)	
Stage (TNM)				*0.027*			*< 0.05*			*< 0.05*
I–II	28 (56%)	9 (32.1%)	19 (67.9%)		–	28 (100%)		–	28 (100%)	
III–IV	22 (46%)	14 (63.6%)	8 (36.4%)		20 (90.9%)	2 (9.1%)		14 (63.8%)	8 (36.2%)	
Tumor depth of invasion				0.999			0.254			0.550
T1–T2	3 (6%)	1 (33.3%)	2 (66.7%)		–	3 (100%)		–	3 (100%)	
T3–T4	47 (94%)	22 (46.8%)	25 (53.2%)		20 (42.6%)	27 (57.4%)		14 (29.8%)	33 (70.2%)	
Lymph node metastasis				0.493			0.503			0.999
Positive	48 (98%)	23 (47.9%)	25 (52.1%)		20 (41.7%)	28 (58.3%)		14 (29.2%)	34 (70.8%)	
Negative	2 (2%)	–	2 (100%)		–	2 (100%)		–	2 (100%)	
Recurrence				*0.013*			*< 0.05*			*< 0.05*
Positive	21 (42%)	14 (66.7%)	7 (33.3%)		20 (95.2%)	1 (4.8%)		14 (66.7%)	7 (33.3%)	
Negative	29 (58%)	9 (31%)	20 (69%)		–	29 (100%)		–	29 (100%)	

Italic values indicate significance of p value (p < 0.05)

### EGFR family expression and prognosis

**Fig. 1:**
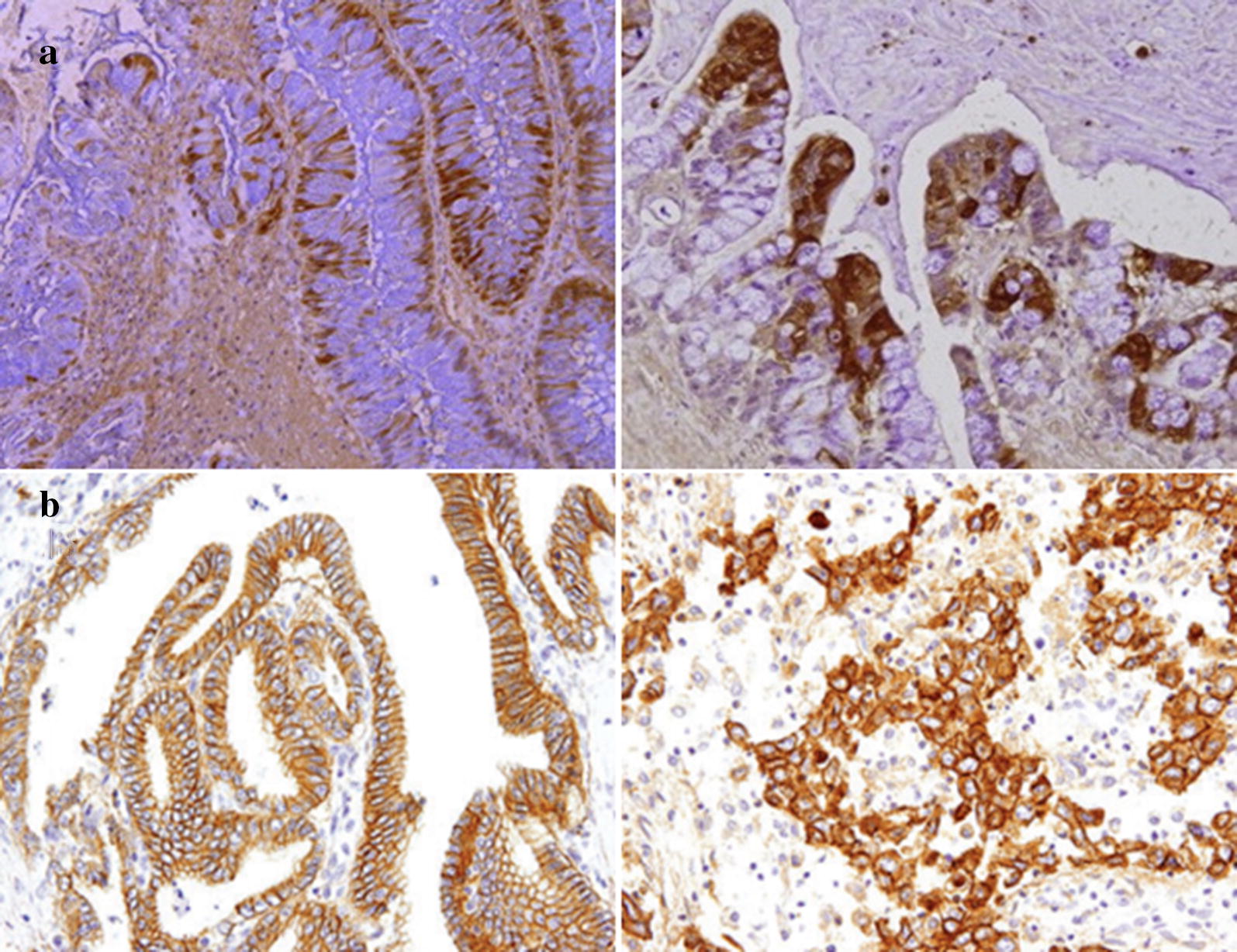
Immunohistochemical staining of ErbB3 in gastric cancer species (**a**). EGFR immunohistochemical staining (**b**)

**Fig. 2:**
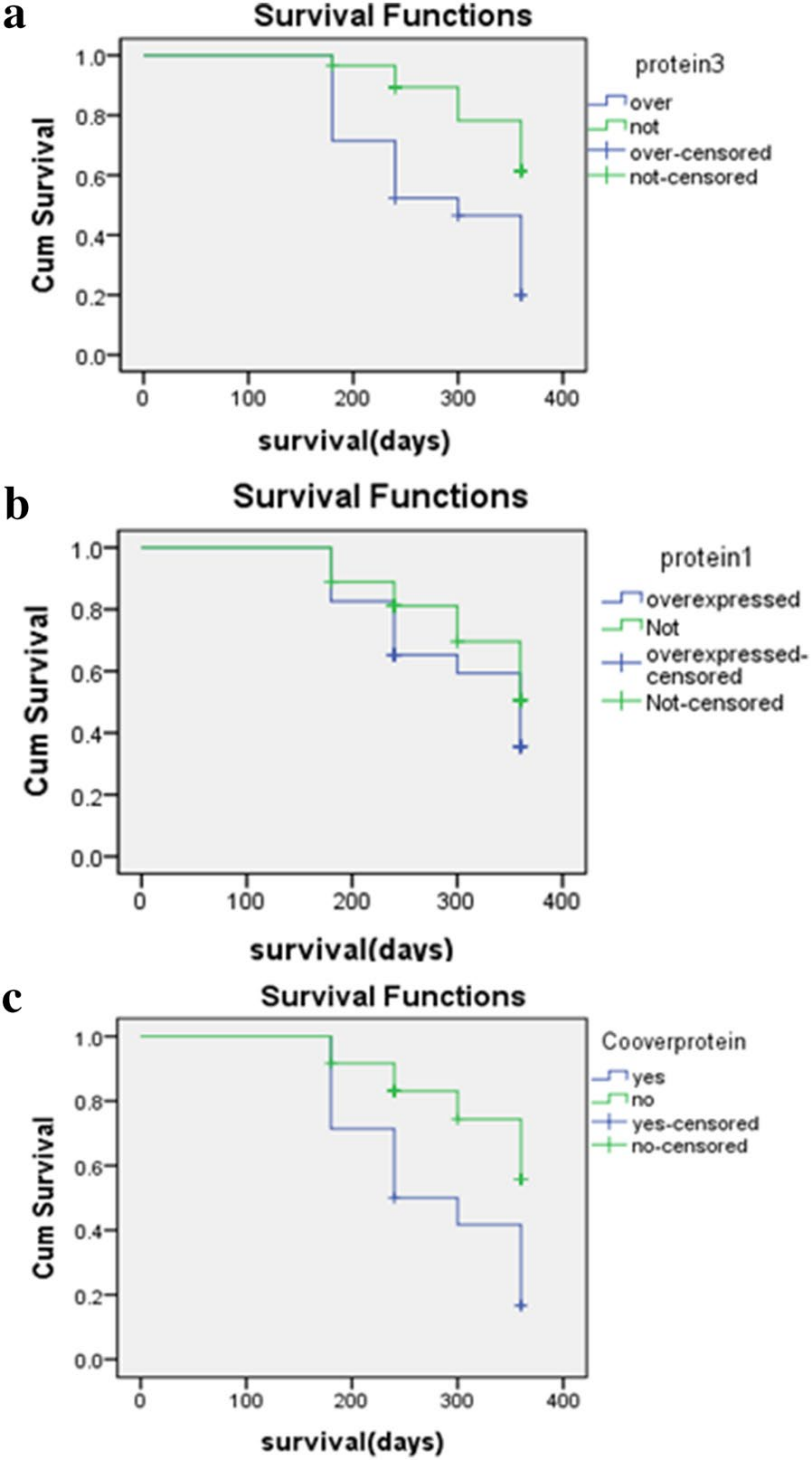
Kaplan-Meier curves for the overall survival of patients with ErbB3 expression (**a**). ErbB1 expression (**b**), ErbB1 and ErbB3 co-overexpression (**c**)

ErbB1 and ErbB3 over expression were observed in 23 (46%) and 20 (40%) of patients, respectively. Fourteen out of 50 (28%) had ErbB1/3 over expression. ErbB1 protein expression was observed in different parts of tumor cells including; membranous (14/50, 28%), cytoplasm (28/50, 56%), and 8 out of 50 (16%) cases had membranous and cytoplasmic expression simultaneously. However, ErbB3 protein expression was observed in cytoplasm (Fig. 1). Seventeen, 19, 20, and 20 out of 20 ErbB3 over expressed cases were infiltrative, poorly differentiated, stage of III/IV, and with a positive tumor relapse (p < 0.05), respectively. ErbB1 protein expression was also significantly correlated with macroscopic features (p = 0.028), tumor differentiation (p = 0.028), recurrence (p = 0.013), and stage of tumor (p = 0.027). There was a significant correlation between ErbB1/3 over expression and grade of tumor in which 13 out of 14 cases (92.9%) were poorly differentiated (p < 0.05). There was a significant correlation between ErbB1/3 over expression and tumor relapse in which all of the cases with such over expression had tumor relapse (p < 0.05). Moreover, there was a significant correlation between ErbB1/3 over expression and stage of tumor in which all of them were in tumor stages of III/IV (p < 0.05). Twelve out of the 14 ErbB1/3 over expressed cases were infiltrative type (p < 0.05). Although, there wasn’t any significant correlation between tumor depth of invasion and ErbB1/3 expression, all of the ErbB1/3 over expressed cases had T3/4 depth of invasion. There was also a significant correlation between tumor size and ErbB3/1 over expression in the level of protein in which the over expressed tumors were smaller than tumors with normal expression (4.25 ± 0.854 vs. 5.14 ± 0.382 cm). In the case of age, it was shown that the ErbB1/3 over expressed cases were older than the other cases (73 ± 6.0 vs. 63 ± 1.8 years old). ErbB3 mRNA over expression was significantly correlated with tumor differentiation (p < 0.05), lymph node involvement (p = 0.001), stage of tumor (p < 0.05), and recurrence (p < 0.05). ErbB1 mRNA over expression was also significantly correlated with ErbB3 protein expression (p = 0.045), tumor differentiation (p = 0.048), and recurrence (p = 0.026). Regarding the log rank test, there wasn’t any significant correlation between ErbB1 protein expression and survival rate (p = 0.267). Whereas, ErbB3 protein expression was significantly associated with poor survival of the patients (p = 0.002). Moreover, the patients who had over expression in both of these markers had a poor survival (p = 0.006) (Fig. 2). The prognostic relevance of ErbB1/3 was evaluated using a multivariate proportional hazard model adjusted for the established clinicopathological features such as histological type, node involvement, tumor location, and differentiation. ErbB3 protein expression (p = 0.046), ErbB1/3 over expression (p = 0.05), histological type (p = 0.018), and lymph node involvement (p = 0.049) were considered as prognostic factors (Table 3). The logistic regression model also showed that the ErbB1/ErbB3 over expression, lymph node involvement, and size of tumor were independent prognostic factors (Table 4).

**Table 3:** Prognostic factors in a multivariate proportional hazard model of the Cox regression

Factors	HR (95% Cl)	p value
Protein ErbB1	9.15 (0.553–33.35)	0.083
Protein ErbB3	0.11 (0.013–0.96)	*0.046*
Intestinal/diffuse	5.47 (1.34–22.34)	*0.018*
Node involvement	1.18 (1.00–1.40)	*0.049*
Tissue differentiation	4.30 (0.553–33.50)	0.163
mRNA ErbB1	1.12 (0.848–1.50)	0.407
mRNA ErbB3	0.90 (0.688–1.15)	0.476
Tumor location	0.54 (0.201–1.48)	0.237
Co-overexpression ErbB1 and ErbB3	0.09 (0.008–1.02)	*0.050*

Italic values indicate significance of p value (p < 0.05)

**Table 4:** Independent Prognostic factors according to Logistic regression model

Factors	**p value**
ErbB1 mRNA	0.061
ErbB3 mRNA	0.461
ErbB1 protein	0.469
ErbB3 protein	0.597
Sex	0.298
Size	*0.050*
Age	0.163
Lymph node involvement	*0.011*
Tumor tissue differentiation	0.318
Stage	0.228
Co overexpression ErbB1 and ErbB3	*0.029*
Tumor location	0.759

Italic values indicate significance of p value (p < 0.05)

## Discussion

Prognostic role of EGFR family is reported in several cancers such as lung, colorectal, and ovarian cancer [17, 30, 38]. Although, the correlation between EGFR overexpression and poor prognosis was observed in many studies, EGFR structural alterations are rare in gastric cancer [39]. It has been shown that, over expression of ErbB1 and ErbB3, and ErbB4 proteins were significantly associated with poor prognosis in gastric cancer patients [2]. EGFR expression has been correlated with shorter overall survival, advanced stages of tumor, and lymph node metastasis in gastric cancer [40, 41]. However, there are some reports about the positive role of EGFR expression in gastric cancer patients and improving the survival of such patients [42]. EGFR has a wide range of expression in gastric cancer ranging from 2 to 44% [41, 43, 44]. Although, HER overexpression has been reported in gastric cancer patients, there is a conflict about the correlation between HER positivity and patient survival [45, 46]. In the present study we assessed the probable correlation between concomitant EGFR and ErbB3 expression and prognosis in gastric cancer. There was a direct correlation between EGFR and ErbB3 expression highlighting that these heterodimers have a significant prognostic role in gastric cancer. It was shown that there were also significant correlations between EGFR/ErbB3 expression and clinicopathological features of patients. However, there was not any correlation between ErbB1 over expression and poor prognosis which is in contrast with some other reports in gastric cancer [40]. We showed that EGFR and ErbB3 co-overexpression is an independent prognostic factor and can also predict the poor survival rate. Although, level of ErbB3 protein expression was correlated with prognosis according to Log-Rank test, there was not any significant correlation between the ErbB1 protein expression and prognosis. In recent studies, it was observed that anti-ErbB2 targeted therapy can cause compensatory overexpression of ErbB3 in breast and colorectal cancers [47, 48]. ErbB2 and ErbB3 co-overexpression decreases survival rate of breast cancer patients [49, 50]. We have observed that there was a significant correlation between ErbB1 and ErbB3 and ErbB3 protein co-overexpression with CA19-9 serum marker. It was observed that co-over expression of these markers have a correlation with higher ages of patients. Moreover, co-over expression was also associated with tumor size in which the over expressed cases had bigger tumors in comparison with the other cases. This refers to the role of ErbB1/3 in early steps of gastric tumors and probably it has not an important role in advanced steps during the gastric cancer progression. Regarding this fact that the ErbB1/3 over expressed cases are in stages of II/IV and have T3/4 depth of invasion, therefore these markers cannot be used as diagnostic markers for the early detection of gastric cancers. Since, all of the ErbB1/3 over expressed cases had metastatic lymph nodes; it seems that these markers can be used for the detection of aggressive type of gastric cancer.

## Conclusion

Co-overexpression of ErbB1 and ErbB3 is accompanied with poor prognosis and can be the cause of resistance to anti-ErbB1/3 therapy. Therefore, targeted therapy inhibits the interaction between ErbB1 and ErbB3 and can be more effective in eradication of tumor progression. Besides, ErbB1 and ErbB3 inhibitors can also prevent the ErbB3 overexpression. Moreover, it seems that the combination of ErbB1/3 can be used as a diagnostic method for the invasive gastric cancer tumors.

## Abbreviations

EGFR: epidermal growth factor receptor
GAPDH: glyceraldehyde 3-phosphate dehydrogenase
